# Adult Hirschsprung disease as acute intestinal obstruction: a case report

**DOI:** 10.11604/pamj.2022.41.11.31148

**Published:** 2022-01-05

**Authors:** Muhammad Ihwan Kusuma, Samuel Sampetoding, Mulkyawan Bahrun, Muhammad Faruk

**Affiliations:** 1 Division of Digestive, Department of Surgery, Faculty of Medicine, Hasanuddin University, Makassar, Indonesia,; 2 Department of Surgery, Dr. Wahidin Sudirohusodo General Hospital, Makassar, Indonesia,; 3 Department of Surgery, Faculty of Medicine, Hasanuddin University, Makassar, Indonesia

**Keywords:** Hirschsprung, adult case, megacolon, bowel obstruction, case report

## Abstract

Most cases of Hirschsprung disease (HD) cases are known in newborns or infants. Nevertheless, some cases with mild symptoms are not identified until acute presentations, such as bowel obstruction present in adolescence or adulthood. We reported a 25-year-old male with a history of chronic constipation from childhood presenting with bowel obstruction due to HD. As an emergency operation, the Hartmann procedure was performed to overcome the obstruction. The histological result showed an aganglionic segment, confirming HD. We plan a definitive Duhamel endorectal pull-through surgery three to six months in the future. Adult HD is uncommon, and clinicians should be aware when patients with histories of chronic constipation from a young age present with intestinal obstruction.

## Introduction

Hirschsprung disease (HD) was first described in 1691 by Frederik Ruysch, a Dutch anatomist and surgeon, as a phenomenon associated with very dilated colon disorders [1,2]. However, the disease was eponymously named after Harald Hirschsprung at the German Pediatric Society conference in Berlin (1886), where Hirschsprung presented cases of two infants who died of complications due to intestinal obstruction [2,3]. HD, another name for congenital aganglionic megacolon, is a disorder characterized by the absence of ganglion cells in the submucosal plexus and myenteric in the bowel segment, which produces a functional obstruction and proximal dilatation of the affected segment [4,5]. Most HD cases are recognized in newborns or infants, but some cases of a milder form can only be identified in teenagers and adults [6]. Here, we report an uncommon case of a male suffering bowel obstruction due to HD.

## Patient and observation

**Patient Information:** a 25-year-old male presented to the emergency department (ED) with a three-day history of abdominal distention. He also complained of flatus and the inability to defecate for three days.

**Clinical findings:** clinical examination revealed vital signs within normal limits. The abdominal region looked distended, bowel contour was seen, auscultation bowel sounds were reduced, and hyper resonant sounds were positive on percussion. Abdominal tenderness was positive. A digital rectal examination (DRE) found an empty vault.

**Timeline of current episode:** he had a history of constipation during the last 20 years, and he had been diagnosed with non-specific proctitis based on lower gastrointestinal endoscopy (LGIE) and biopsy results. His constipation had worsened four months ago, but it was relieved when he consumed laxative drugs. The patient had not undergone any previous surgery or hematochezia, and he had no family history of HD.

**Figure 1:**
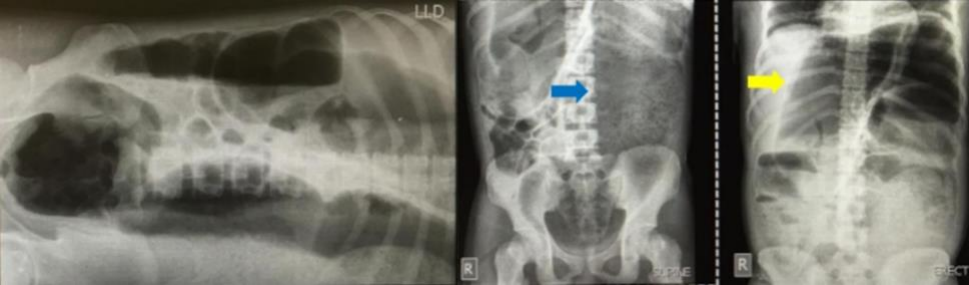
an X-ray of plain abdomen 3 views showed stool impaction (blue arrow) and dilated colon (yellow arrow); the colon diameter was more than 10 cm

**Diagnostic assessment:** laboratory tests showed slightly increased leukocytes 10.600 cell/ml with a 50% neutrophil composition. The patient was negative for the hepatitis B virus. The abdominal X-ray showed dilatation of the colon and absence of air distribution in the rectum with a lot of fecal material, indicating colonic volvulus (Figure 1).

**Diagnosis:** we diagnosed the patient with bowel obstruction, which we suspected was due to volvulus.

**Figure 2:**
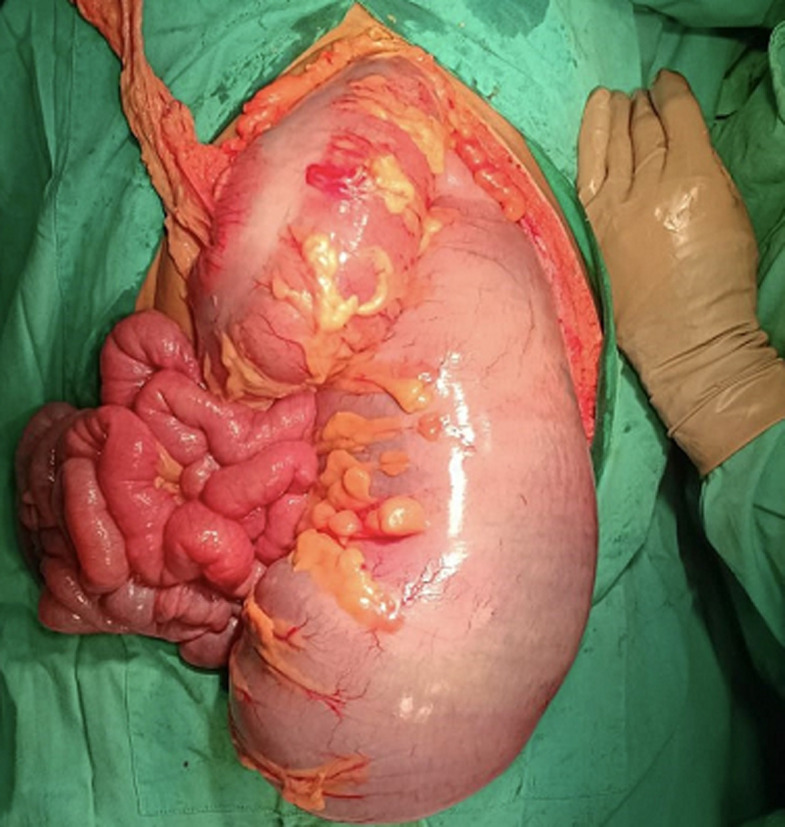
a colon appeared extremely dilated (diameter more than 10 cm) protruded while opening the abdomen

**Therapeutic interventions:** the patient was transferred to the operating room two hours after arriving in the ED, and we performed an exploratory laparotomy. Immediately after the midline incision, the colon appeared extremely dilated and congested upstream of the ileocecal junction. The obstruction observed on the rectosigmoid was about 50 cm long (Figure 2). During surgical exploration, no tumors or adhesions were found. A Hartmann procedure was performed, and the resected rectosigmoid was sent to the pathologist.

**Figure 3:**
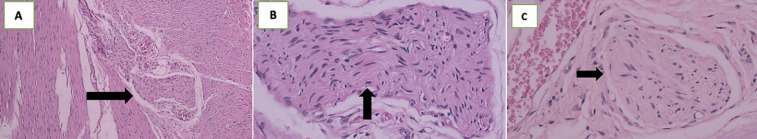
(A,B,C) histopathology slide showing hypertrophic nerve bundles and the absence of ganglion cells (arrows) (hematoxylin and eosin staining at 10x, 40x, and 100x magnification)

**Follow-up and outcome of interventions:** the pathologist's report showed an aganglionic segment, confirming Hirschsprung's disease (Figure 3). The patient fully recovered and was discharged on a postoperative day five with a functional colostomy in situ. We plan a definitive Duhamel endorectal pull-through surgery three to six months in the future.

**Patient perspective:** the patient shared their perspective on the treatment in defecation with a functional colostomy stating that they felt no symptoms and could return to their normal activity.

**Informed consent:** the patient provided informed consent for the publication of his clinical data. The presented data are anonymized, and the risk of identification is minimal.

## Discussion

We report the case of an adult with HD whose first clinical symptoms involved signs of bowel obstruction. An emergency laparotomy was performed, and we identified an obstruction in the rectosigmoid, which required the Hartmann procedure. HD, or congenital aganglionic megacolon, is a condition that involves a functional intestinal obstruction in part or all of the colon at the aganglionic level [7]. HD occurs due to the failure of cephalocaudal migration of ganglion cells in the 12^th^ week of pregnancy [8]. Identification of Hirschsprung disease in adulthood is uncommon [9,10]. According to the level of colon involvement, HD is categorized into six types: 1) all of the rectal and sigmoid colons is affected (common type); 2) only the terminal rectal area is affected (ultrashort segment type); 3) from the rectal terminal to the ampulla is affected (short segment type); 4) from the rectum to the descending colon and transverse colon are affected (long segment type); 5) from the rectum, the entire colon and the last 10 cm of the terminal ileum are affected (total colonic type); and 6) the entire intestinal segment can further be affected based on the type of total colon (total bowel type) [10].

The clinical symptoms of patients with adult HD are described as constipation experienced from birth [11]. They rarely come to emergency departments with late symptoms such a bowel obstructions, as in our case. According to Hsieh *et al*.(2006) and Qiu *et al*.(2013), the first documented case of adult HD was reported by Rosin *et al*.(1950); the patient was a 54-year-old male with a short aganglionic segment [12,13]. HD incidence is around 1 in 5,000 live births and is often present in the neonatal period [4]. It can occur alone or in combination with other developmental disorders. About 10-12% of all HD cases occur in children with Down syndrome.

Approximately 15% of HD cases have family origins, but the majority are sporadic. Based on the genetic examination, about 15% of sporadic cases and half of the family cases are related to the inactivation of RET receptor gene mutations for a tyrosine kinase (TK) on chromosome 10q, and a small proportion of cases are related to endothelin-B receptor mutations [14]. In our case, the patient has no familial history or symptoms of Down syndrome. The incidence of adult HD is only 300 cases before 2016 [6,10], with males predominating over females (4: 1) [4,8]. The ages of adult HD patients range from 14 to 70 years, with an average age of 23.9 years. The majority of cases involve patients under 30 years of age [6].

Adult patients with HD usually present with a history of chronic/refractory constipation (73-92%), abdominal distension (83-86%), frequent palpable fecal mass (50-56%), fecal impaction (25-36%), and a history of using enemas regularly to defecate (73-92%) [4,12]. Approximately 5% of patients with minimal disease symptoms may not be diagnosed until early adulthood (in the second and third decades of life). They are often misdiagnosed with chronic constipation. HD may not be diagnosed until acute symptoms with complications such as obstructive colitis or sigmoid volvulus occur [4,10]. Clinical symptoms such as abdominal distension begin to appear at a young age in most patients, but most patients do not undergo treatment because the symptoms are mild [10]. These symptoms often get worse as the patient ages. Bowel obstruction is the main symptom nowadays [4,10]. HD cases in adults are uncommon, and when found in this age group, it appears as the ultrashort segment HD type [14]. This case presented with a bowel obstruction in the rectosigmoid with a history of chronic constipation beginning at a young age.

HD diagnostic investigations include anorectal manometry, barium enema, and rectal biopsy [10,14]. On examination of the barium enema in classic HD cases, proximal megacolon and anorectal narrowing can be found. In this case, histopathologic results from the rectal biopsy in the distal bowel showed increased acetylcholinesterase (AChE) from nerve fibers and the absence of ganglion cells. The gold standard for definitive diagnosis of HD is a rectal biopsy, which shows aganglionic presence in the myenteric plexus and hypertrophy of these nerve endings [6]. HD diagnosis was made by considering clinical symptoms, history of illness, laboratory examination, and histopathological examination [10].

A two-stage surgical procedure is recommended as the safest management for treating adult HD. This is because chronic excess stool causes massive colonic dilatation and disparity in the bowel lumen, which results in difficulty performing bowel anastomosis [14]. The first stage of surgical management involves frozen section biopsy and colostomy, followed by abdominal pull-through a few months later. This allows the large intestine to gradually return to its normal condition in the time interval between the first and second operations and also reduces the risk of postoperative complications [14]. Six surgical methods can be used for adult HD: the Duhamel procedure, the Swenson procedure, the Soave procedure, myectomy, low anterior resection (LAR) combined with myectomy, and LAR combined with colectomy [4,15].

In a systematic review, Doodnath *et al*. (2010) reported that the Duhamel procedure is the most frequently applied in the world [16]. The basic principle of this procedure is to pull the proximal colon towards the anus through the posterior aganglionic part of the rectum, joining the aganglionic posterior wall of the rectum with the anterior wall of the proximal ganglionic colon to form a new cavity with end-to-side anastomosis. Duhamel's surgical procedure has lower operative morbidity than the Soave and Swenson procedures. Few pelvic dissections are performed to complete the Duhamel procedure, so the risks of pelvic nerve injury, impotence rate, and anastomotic dehiscence are minimal [15]. The advantage of myomectomy is that it is technically more accessible and has lower morbidity. Still, it has worse functional outcomes and can only be used in the ultrashort segment HD type [4]. In our case, after the emergency Hartman sigmoid colostomy, we considered the definitive treatment of our patient with the Duhamel procedure. However, the choice of technique depends on the experience and habits of the surgeons.

Untreated adult HD patients are mostly at high risk of mortality due to complications such as perforation, intestinal obstruction, enterocolitis, malnutrition, and dehydration [11]. Complications arising in adults who have had surgery for HD are anastomotic dehiscence, enterocolitis, fistula-in-ano, retraction of the colon, pelvic abscess, impotence, anastomotic stricture, anemia, peri-anal abscess, pulmonary embolism, and ischemia at the anastomotic site [14].

## Conclusion

Hirschsprung disease (HD) is an uncommon entity in adulthood, and clinicians should be aware if patients present with intestinal obstruction and have had a history of chronic constipation since younger age.
